# Childbirth outcomes and ethnic disparities in Suriname: a nationwide registry-based study in a middle-income country

**DOI:** 10.1186/s12978-020-0902-7

**Published:** 2020-05-07

**Authors:** Kim J. C. Verschueren, Zita D. Prüst, Raëz R. Paidin, Lachmi R. Kodan, Kitty W. M. Bloemenkamp, Marcus J. Rijken, Joyce L. Browne

**Affiliations:** 1 Division Women and Baby, Department of Obstetrics, Birth Centre Wilhelmina’s Children Hospital, Utrecht, The Netherlands; 2 Anton de Kom University, Paramaribo, Suriname; 3 Department of Obstetrics and Gynaecology, Diakonessenhuis, Paramaribo, Suriname; 4 Department of Obstetrics and Gynaecology, Academical Hospital Paramaribo, Paramaribo, Suriname; 5 Julius Global Health, The Julius Centre for Health Sciences, University Medical Centre Utrecht, Utrecht, The Netherlands

**Keywords:** Perinatal registry, Maternal mortality, Stillbirths, Middle-income country, Suriname, Ethnicity, Racial, Disaggregate

## Abstract

**Background:**

Our study aims to evaluate the current perinatal registry, analyze national childbirth outcomes and study ethnic disparities in middle-income country Suriname, South America.

**Methods:**

A nationwide birth registry study was conducted in Suriname. Data were collected for 2016 and 2017 from the childbirth books of all five hospital maternity wards, covering 86% of all births in the country. Multinomial regression analyses were used to assess ethnic disparities in outcomes of maternal deaths, stillbirths, teenage pregnancy, cesarean delivery, low birth weight and preterm birth with Hindustani women as reference group.

**Results:**

18.290 women gave birth to 18.118 (98%) live born children in the five hospitals. Hospital-based maternal mortality ratio was 112 per 100.000 live births. Hospital-based late stillbirth rate was 16 per 1000 births. Stillbirth rate was highest among Maroon (African-descendent) women (25 per 1000 births, aOR 2.0 (95%CI 1.3–2.8) and lowest among Javanese women (6 stillbirths per 1000 births, aOR 0.5, 95%CI 0.2–1.2). Preterm birth and low birthweight occurred in 14 and 15% of all births. Teenage pregnancy accounted for 14% of all births and was higher in Maroon women (18%) compared to Hindustani women (10%, aOR 2.1, 95%CI 1.8–2.4). The national cesarean section rate was 24% and was lower in Maroon (17%) than in Hindustani (32%) women (aOR 0.5 (95%CI 0.5–0.6)). Cesarean section rates varied between the hospitals from 17 to 36%.

**Conclusion:**

This is the first nationwide comprehensive overview of maternal and perinatal health in a middle income country. Disaggregated perinatal health data in Suriname shows substantial inequities in outcomes by ethnicity which need to be targetted by health professionals, researchers and policy makers.

## Plain English summary

In middle-income country Suriname we studied all hospital births to describe childbirth outcomes and to discover inequities by ethnicity. In 2016 and 2017, 18.290 women gave birth to a baby in either of the five hospitals, which represents 86% of all national births in Suriname. There were 20 maternal deaths, resulting in a maternal mortality ratio of 112 per 100.000 live births. There were 285 stillbirths beyond 28 weeks of gestation, resulting in a late stillbirth rate of 16 per 1000 births. Stillbirth rate was highest in Maroon women (25 per 1000 births) and lowest in Javanese women (6 per 1000 births). Teenage pregnancies accounted for 14% of all births, was highest in Indigenous (21%) and Maroon (18%) women, and lowest in Hindustani (10%) and Chinese (3%) women. Babies with low birth weight accounted for 15% of all births and were most frequently seen in Hindustani women (18%). The cesarean section rate was 24%, but varied from 17 to 36% between the five hospitals. Hindustani women were almost twice more likely to receive a cesarean section than Maroon women (32 vs. 17%). Disaggregating perinatal health data is encouraged to identify and help target inequity within the health system. In conclusion, there are substantial inequities by ethnicity with Maroon women experiencing the highest risk on adverse outcomes (maternal mortality, stillbirth, increased preterm birth, low apgar score). The inequitable access to care experienced by women of African-descent requires policy makers to review possible interventions.

## Background

Maternal and perinatal vital registration systems are essential to monitor outcomes of pregnant women and their offspring, identify inequities in service provision and health outcomes and facilitate quality control in perinatal care [1, 2]. Following lessons learned from the Millennium Development Goals (MDGs), the Sustainable Development Goals (SDGs) call for statistics “disaggregated by income, gender, age, race, ethnicity, migratory status, disability, geographic location and other characteristics relevant in national contexts” to monitor progress and identified inequities in health outcomes [2–4].

The *Global Strategy for Women’s, Children’s and Adolescents’ Health, 2016–2030* is a roadmap for ending all preventable maternal and newborn deaths (including stillbirths) and is central to the achievement of the SDGs [5]. This strategy urgently calls for the extension and strengthening of health information systems to generate high quality data and evidence to measure progress and be able to reach the target of a global maternal mortality ratio (MMR) under 70 per 100.000 live births and stillbirth rate (SBR) under 12 stillbirths per 1000 births [2, 6].

National childbirth registries have been established in several high income countries [7–9]. Low and middle-income countries (LMIC) are increasingly investing in robust national information on maternal and perinatal health indicators for SDG monitoring. However, given the complexity of establishing a well-functioning registry system, data collection in these countries is often not uniform, lack important indicators and data are frequently missing [1]. In many (currently, 34) Latin American and Caribbean countries, the Perinatal Information System (SIP), a digital clinical record and local management software standard has been implemented [10]. Suriname, an upper middle-income country, is one of the countries where SIP will be re-launched after a previous attempt in 2014, which failed for unknown reasons [11].

Ethnic disparities in birth outcomes have modestly been studied compared to other social determinants of health, such as wealth, education, age and place of residence. Several studies in high-income countries have shown that women of African descent experience a two to six times higher risk for severe maternal outcomes compared to Caucasian women (often linked to socio-economic factors) [12–16]. Suriname is particularly of interest as it has multiple ethnic groups without one great majority [17].

To promote an efficient and adequate implementation process of the new perinatal data system in Suriname and subsequently evaluate its effect, this study provides a baseline assessment of perinatal data from all hospitals in the country. Our study aims to evaluate the current perinatal registry, analyze national maternal and perinatal characteristics and study ethnic disparities in childbirth outcomes in Suriname.

## Methods

### Study design

A two-year registry-based nationwide study of all hospital births was conducted, using the childbirth books of the five maternity wards between January 1st, 2016 and December 31st, 2017.

### Study context and ethnicities

Suriname is a multi-ethnical, upper middle-income country on the northeast coast of South-America. With an estimated population of 598,000 people, it is one of the least populous countries in the Americas [18, 19]. Ethnical distribution among the general Surinamese population in 2013 was: Hindustani (27%), Maroon (22%), Creole (16%), Javanese (14%), Mixed (13%), Indigenous (4%), Chinese (1%) and Other (3%) [19, 20]. Diversity in Suriname is a reflection of the country’s history. Indigenous people, also known as Amerindians, are the original inhabitants of the country. Maroon and Creoles are African-descendants who were enslaved and brought to Suriname in the seventeenth and eighteenth century. Maroon people, in contrast to Creoles, escaped into the interior of the country. Creoles gained their freedom in 1863 when slavery was abolished in Suriname and often have mixed African - European (Dutch and British) ancestry. Asian-descendants: Hindustani (from East-India), Javanese (from Indonesia, then a Dutch-ruled colony) and Chinese people, came to Suriname in the late nineteenth century as contract workers. Mixed people are the result of interchanging identities between almost all ethnicities. Other ethnicities include mostly Brazilians, Caucasians (descendants of Dutch colonists) and few Lebanese [17].

### Study setting

The study was conducted in all hospitals in Suriname: four hospitals located in the capital Paramaribo and one hospital in Nickerie (West coast). Institutions perform approximately 92% of all births in Suriname with approximately 86% in hospitals and 6% in primary health care centers. Information regarding the primary health care births, home births (4%) and births of unknown location (4%) were not available [19, 20].

The Multiple Indicator Cluster Survey (MICS) of 2018, estimated that 13% of women did not receive antenatal care, 56% received their first antenatal care visit within the first 3 months of pregnancy, 62–80% received at least four antenatal care visits and 95–98% of births were attended by skilled birth attendants [20]. Maternal mortality has dropped 43%, from 226 to 130 per 100.000 live births, between 1990 and 2015, as demonstrated by two Reproductive Age Mortality Surveys [21, 22].

### Participants and data collection

All hospital births with babies born by at least 22 weeks of gestation or with a birth weight of at least 500 g, were eligible for inclusion [23]. Paper childbirth books provide manually written data on every birth in all hospitals. Birth attendants are responsible for the information to be registered in the books. Hospital personnel digitalized this data in Microsoft Excel and IBM SPSS with instructions and prespecified definitions. All variables were entered in the secured database anonymously. Three of the authors (KV, ZP, RP) cross-checked the digital data with the paper childbirth book data. Medical files were assessed for stillbirths and maternal deaths to validate the death.

### Variables

The digitalization, software used and reported variables per hospital can be found in Table 1 and an elaborated version in Additional file 1. Ethnicity was self-reported similar to the MICS [20]. Teenage pregnancy was defined as childbirth below the age of 20 years [24]. Severe anemia was defined according to the WHO definition: a hemoglobin level below 70 g/L (4.3 mmol/L) was considered severe and below 100 g/L (6.2 mmol/L) was moderate [25]. Sickle cell anemia is assessed during antenatal care in each woman in Suriname, but not reported in the childbirth book and could therefore not be studied. Outcome variables were coded into categories, based on the Dutch Perinatal data registry data [26]. Preterm birth was defined as childbirth before 37 weeks of gestation [26]. Low birth weight was defined as a newborn with a birth weight below 2500 g [26]. APGAR score was considered low when the 5 min APGAR score was below 7 [26]. Postpartum hemorrhage (PPH) was defined as blood loss of at least 500 mL and severe PPH at least 1000 mL [27]. Stillbirth was defined as a fetus born with no signs of life. Late stillbirth was defined, according to the International Classification of Diseases – Perinatal Mortality (ICD-PM), as stillbirth after 28 weeks of gestation or, if gestational age was unknown, birth weight of 1000 g or more [28]. Stillbirth rate (SBR) was calculated as the number of late stillbirths per 1000 births beyond 28 weeks of gestation (or > 1000 g) [28]. Maternal death was defined according to the International Classification of Diseases – Maternal Mortality (ICD-MM), as death of a woman while pregnant or within 42 days of termination of pregnancy, from any cause related to or aggravated by the pregnancy or its management but not from accidental or incidental causes [29]. The national MMR was calculated as the number of maternal deaths per 100.000 live births beyond 22 weeks of gestation (or > 500 g) [29]. Data on social economic status such as income and level of education, and data on obesity and smoking were not available.

**Table 1:** Overview of maternal and perinatal data registration per hospital in Suriname

	Hospital				
	I	II	III	IV	V
**Childbirth registry**	On paper	On paper	On paper	On paper	On paper
**Digitalizing of paper parturition books**	Special secretary for this task	General secretary, with other responsibilities	General secretary, with other responsibilities	No one; students used for this study	Responsible midwife doing the delivery
**Software used**	SPSS	Excel	Access	Excel	Excel
**Common reported variables**	Maternal age, ethnicity, gravidity, parity, gestational age, singleton or twin, data and time of delivery, presentation, mode of delivery, indication for cesarean section, APGAR score after 1 and 5 min, sex, birth weight, length, head circumference, stillbirth, weight placenta, length of umbilical cord, rupture type, blood loss				
**Other variables reported per hospital**					
HIV	Yes	–	–	Yes	Yes
Hepatitis B	–	–	–	Yes	–
Syphilis	–	–	–	Yes	–
1st antenatal care visit	Yes	–	–	Yes	–
Amount of ANC visits	–	–	Yes	–	–
1st ultrasound	Yes	–	–	–	–
Hemoglobin level	Yes	–	–	Yes	Yes
Blood type and rhesus	Yes	Yes	–	Yes	Yes
Induction of labor	Yes	–	–	–	–
Augmentation of labor	Yes	–	–	–	–
Duration 3rd stage of labor	Yes	–	–	–	–
Active 3rd stage of labor	–	Yes	Yes	Yes	Yes

### Statistical methods

Descriptive statistics were used for baseline characteristics analysis using frequencies and percentages for categorical data. Differences between groups were tested with chi-square test for significance (*p* < 0.05). Missing data on ethnicity was negligible (< 5%) and no data imputation was performed. To assess the relation between ethnic groups and health outcomes, multinomial logistic regression was performed. Hindustani ethnicity was used as reference group as they represent the largest proportion of the general population. Odds ratios (OR) were obtained, which were interpreted as relative risks in the case that the probability of the disease was less than 10% [30]. Possible confounders were selected by constructing causal diagrams and adjusted odds ratios (aOR) were obtained [31]. IBM SPSS version 25 was used for statistical analyses.

### Ethical approval

This research was performed according to the Declaration of Helsinki and has been approved by the ethical review board of the Surinamese Committee on Research Involving Human Subjects (#VG11–18).

## Results

There were 18.290 hospital births, 9202 in 2016 and 9088 in 2017. A total of 18.504 babies were born, 97.9% (*n* = 18.118) were live births.

The approach to maternal and perinatal data registration by the different hospitals is shown in Table 1. Additional file 2 illustrates maternal and neonatal characteristics and outcomes per hospital. The largest differences between the hospitals are teenage pregnancy rates (range from 7.3 to 17.9%), ethnical distribution, grand multiparous births (range from 4.6 to 18.5%), low birth weight rates (range from 11.3 to 20.8%), cesarean section rates (range from 17.4 to 36.2%) and stillbirth rates (range from 7.3 to 25.6 per 1000 births).

**Fig. 1:**
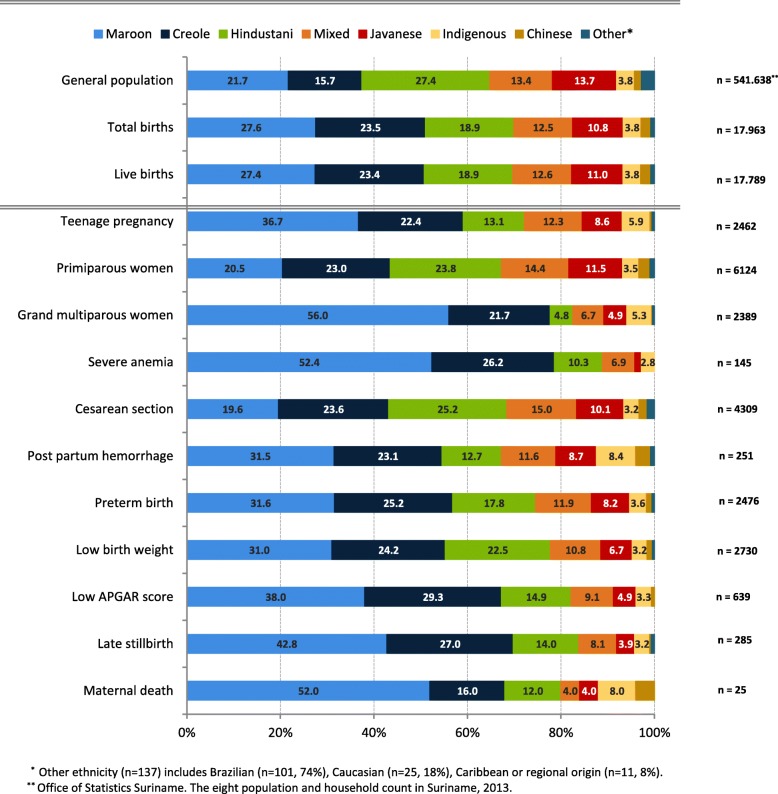
Ethnic distribution in percentages among the pregnant population

Table 2 presents maternal and perinatal characteristics. Median age for nulliparous women to give birth was 22 (IQR 19–27) years. Teenage pregnancies occurred in 13.8% (*n* = 2518) of births. The majority of women giving birth were African descendants: Maroon (27.6%) and Creole (23.5%) women (see Fig. 1). Preterm birth occurred in 14.0% (*n* = 2529) and birthweight was below 2500 g in 15.1% (*n* = 2774). Cesarean section was performed in 24.1% of all births. In primiparous women the cesarean section rate was 27.0% (*n* = 1683). Repeat cesareans (*n* = 1321) contributed to 30% of all cesarean sections. Post partum hemorrhage of 500 mL or more occurred in 7.9% of births (*n* = 1287/16348). In cesarean sections the incidence of post partum hemorrhage was 17.9% (*n* = 501/2838, missings *n* = 1571) and in vaginal births 5.8% (*n* = 786/13510, missings *n* = 371).

**Table 2:** Maternal characteristics of all hospital births in Suriname in 2016 and 2017

	Total *n* = (%)
**Total deliveries**	18,290
**Hospitals**	
I	4380 (23.9)
II	5070 (27.7)
III	5089 (27.8)
IV	3034 (16.6)
V	717 (3.9)
**Age** (years)	
< 20	2518 (13.8)
20–35	13,646 (74.8)
> 36	2087 (11.4)
*Missings n = 39*	
**Ethnicity**	
Maroon	4950 (27.6)
Creole	4217 (23.5)
Hindustani	3395 (18.9)
Mixed	2254 (12.5)
Javanese	1948 (10.9)
Indigenous / Amerindian	681 (3.8)
Chinese	381 (2.1)
Other^a^	137 (0.8)
*Missings n = 327*	
**Parity**	
0	6243 (34.3)
1–3	9553 (52.4)
> 4	2428 (13.3)
*Missings n = 66*	
**Gestational age (GA)**	
< 28 weeks	217 (1.2)
28–36 weeks	2312 (12.8)
> 37 weeks	15,569 (86.0)
*Missings n = 192*	
**Anemia**	
Moderate	2571 (39.9)
Severe	153 (2.4)
*Missings n = 11.851*	
**Multiple pregnancy**	
Twin	208 (1.1)
Triplet	3 (−)
**Mode of delivery**	
Spontaneous	13,650 (74.6)
Instrumental	231 (1.3)
Cesarean section	4409 (24.1)
**Presentation**	
Cephalic	17,671 (96.6)
Breech	560 (3.1)
Transverse	59 (0.3)
**Post partum hemorrhage**	
500–999 mL	1031 (6.3)
> 1000 mL	256 (1.6)
*Missings n = 1942*	
**Total babies born**	18,504
Live births	18,118 (97.9)
Stillbirths, > 22 weeks	386 (2.1)
**Sex**	
Girls	8915 (48.2)
*Missings n = 17*	
**Birth weight**	
< 2500 g	2774 (15.1)
2500–4000 g	15,069 (81.9)
> 4000 g	558 (3.0)
*Missings n = 103*	
**Apgar-score 5 min**	
Below 7	648 (3.9)
*Missings n = 1704*	
**Late stillbirth**	
n=	285
SBR per 1000 hospital births ^b^	15.6
**Maternal deaths**	
n=	25^c^
National MMR per 100.000 LB ^d^	127
n=	20
Hospital-based MMR per 100.000 LB ^d^	110

^a^Ethnicity other: Brazilian (n = 101, 0.5%), Caucasian (*n* = 25, 0.1%), Caribbean (*n* = 11)

^b^SBR: stillbirth rate = per 1000 births > 28 weeks or > 1000 g

^c^Two maternal death occurred at home, one during transport, two in primary health care services. Four deaths occurred antepartum, three of which in the hospitals

^d^MMR: maternal mortality rate, maternal deaths per 100.000 live births (LB)

Twenty maternal deaths occurred in the hospitals, resulting in a hospital-based maternal mortality ratio (MMR) of 112 per 100.000 live births. Among all babies with a gestational age of > 22 weeks or more (or if unknown, > 500 g), 386 stillbirths (2.1%) occurred. Of these, 285 were late stillbirths (gestational age > 28 or if unknown > 1000 g), resulting in a hospital-based stillbirth rate of 15.6 per 1000 births.

Table 3 and Fig. 1 show ethnic disparities in maternal and perinatal characteristics. Table 4 presents associations between ethnic group and outcomes, adjusted for confounders and with Hindustani women as reference group. Teenage pregnancy was twice higher among Maroon women (18.3%, *n* = 904) compared to Hindustani women (9.5%, *n* = 322), (aOR 2.1, 95%CI 1.8–2.4). Compared to Hindustani women, Maroon women had a higher risk for severe anemia (4.9%, aOR 2.4, 95%CI 1.4–4.3). Maroon and Indigenous women were most often grand multiparous women (27.1%, aOR 14.4 (95%CI 11.9–18.1) and 19%, aOR 9.9 (95%CI 7.4–13.4)) compared to Hindustani (3.4%). Women of other ethnicities had a significantly lower risk of giving birth to low birth weight infants compared to Hindustani women (aOR range of 0.4–0.7). The cesarean section rate was lower in Maroon women (17.1%) compared to Hindustani women (32.1%) (aOR 0.7 (95%CI 0.6–0.8)). Post-partum hemorrhage occurred most frequently in Indigenous women (8.1%, aOR 3.0, 95%CI 1.7–5.3).

**Table 3:** Ethnic disparities in maternal and perinatal characteristics and outcomes in Suriname, 2016–2017

	Total *n* = (%)	Maroon *n* = (%)	Creole *n* = (%)	Hindustani *n* = (%)	Mix *n* = (%)	Javanese *n* = (%)	Indigenous *n* = (%)	Chinese *n* = (%)	Other ^**a**^ *n* = (%)	***p***-value
**Total births**	18,290	4950 (27.6)	4217 (23.5)	3395 (18.9)	2254 (12.5)	1948 (10.8)	681 (3.8)	381 (2.1)	137 (0.8)	
**Maternal age**										
< 20 years	2462 (13.7)	904 (18.3)	552 (13.1)	322 (9.5)	303 (13.5)	211 (10.9)	146 (21.4)	11 (2.9)	13 (9.5)	
20–35 years	13,420 (74.9)	3383 (68.4)	3164 (75.1)	2774 (82.0)	1704 (75.8)	1504 (77.4)	465 (68.3)	326 (85.6)	101 (73.7)	**< 0.05**
> 36 years	2043 (11.4)	654 (13.2)	496 (11.8)	286 (8.5)	241 (10.7)	229 (11.8)	70 (10.3)	44 (11.5)	23 (16.8)	
**Parity**										
0	6124 (34.2)	1253 (25.4)	1411 (33.6)	1456 (43.0)	883 (39.3)	704 (36.2)	212 (31.3)	148 (38.8)	57 (41.6)	
1–3	9387 (52.5)	2346 (47.5)	2267 (54.0)	1812 (53.6)	1204 (53.6)	1122 (57.7)	338 (49.9)	228 (59.8)	71 (51.8)	**< 0.05**
> 4	2389 (13.3)	1338 (27.1)	519 (12.4)	115 (3.4)	159 (7.1)	117 (6.0)	127 (18.8)	5 (1.3)	9 (6.6)	
**Gestational age**										
< 28 weeks	210 (1.2)	72 (1.5)	64 (1.5)	35 (1.0)	21 (0.9)	10 (0.5)	7 (1.0)	1 (0.3)	–	
28–37 weeks	2266 (12.7)	711 (14.6)	561 (13.4)	405 (12.0)	274 (12.3)	192 (9.9)	82 (12.2)	29 (7.7)	13 (9.7)	**< 0.05**
Term	15,299 (86.1)	4104 (84.0)	3548 (85.1)	2933 (87.0)	1933 (86.8)	1728 (89.5)	583 (86.8)	349 (92.1)	121 (90.3)	
**Anemia**_antepartum_										
Moderate	2258 (36.4)	808 (51.8)	667 (43.8)	372 (33.5)	146 (20.9)	147 (19.0)	71 (37.6)	36 (13.9)	11 (11.7)	**< 0.05**
Severe	145 (2.3)	76 (4.9)	38 (2.5)	15 (1.4)	10 (1.4)	2 (0.3)	4 (2.1)	–	–	
**Mode of delivery**										
Spontaneous	13,428 (74.8)	4074 (82.3)	3174 (75.3)	2223 (65.5)	1575 (69.9)	1482 (76.1)	531 (78.0)	301 (79.0)	69 (50.4)	**< 0.05**
Vacuum	225 (1.3)	31 (0.6)	28 (0.7)	84 (2.5)	34 (1.5)	31 (1.6)	12 (1.8)	4 (1.0)	1 (0.7)	
Cesarean	4309 (24.0)	845 (17.1)	1015 (24.1)	1088 (32.1)	645 (28.7)	435 (22.3)	138 (20.3)	76 (19.9)	67 (48.9)	
**Presentation**										
Cephalic	17,359 (96.7)	4787 (96.7)	4093 (97.1)	3252 (95.8)	2193 (97.3)	1873 (96.1)	661 (97.1)	367 (96.3)	134 (97.8)	**0.03**
**Post partum hemorrhage**										
500–999 mL	1012 (6.3)	288 (6.3)	239 (6.3)	140 (4.8)	144 (7.3)	121 (6.9)	50 (8.1)	21 (6.2)	9 (9.0)	**< 0.05**
> 1000 mL	251 (1.6)	79 (1.7)	58 (1.5)	32 (1.1)	29 (1.5)	22 (1.3)	21 (3.4)	8 (2.4)	2 (2.0)	
**Total babies born**	18,504	5035 (27.7)	4268 (23.5)	3422 (18.8)	2277 (12.5)	1965 (10.8)	687 (3.8)	382 (2.1)	139 (0.8)	
Live Births	17,789 (97.9)^b^	4877 (96.9)	4157 (97.4)	3370 (98.5)	2240 (98.4)	1951 (99.3)	674 (98.1)	381 (99.7)	139 (98.6)	**< 0.05**
Stillbirths (> 22 weeks or 1000 g)	386 (2.1)	158 (3.1)	111 (2.6)	52 (1.5)	37 (1.6)	14 (0.7)	13 (1.9)	1 (0.3)	0 (0)	
**Sex**										
Girl	8767 (48.3)	2485 (49.4)	2083 (48.9)	1637 (47.9)	1064 (46.8)	926 (47.1)	321 (46.9)	181 (47.4)	70 (50.4)	0.39
**Birth weight**										
< 2500 g	2730 (15.1)	847 (16.9)	662 (15.6)	614 (18.0)	294 (13.0)	183 (9.4)	87 (12.6)	32 (8.4)	11 (8.0)	
2500–4000 g	14,805 (81.9)	4047 (80.8)	3449 (81.4)	2710 (79.6)	1887 (83.2)	1690 (86.4)	568 (83.4)	334 (87.9)	120 (86.1)	**< 0.05**
> 4000 g	542 (3.0)	115 (2.3)	127 (3.0)	81 (2.4)	88 (3.9)	83 (4.2)	26 (3.8)	14 (3.7)	8 (5.8)	
**APGAR 5 min’**										
< 7	639 (3.8)	243 (5.3)	187 (4.8)	95 (3.0)	58 (2.8)	31 (1.7)	21 (3.5)	4 (1.1)	–	**< 0.05**
**Late stillbirth**										
n=	285	122	77	40	23	11	9	1	2	**< 0.05**
SBR per 1000 births ^c^	15.6	25.0	18.6	11.9	10.3	5.7	13.4	–	14.6	
**Maternal deaths**										
Nationwide maternal deaths, n=	25	13	4	3	1	1	2	1	–	
MMR per 100.000 national live births ^d^	127	N/A	N/A	N/A	N/A	N/A	N/A	N/A	N/A	
Hospital maternal deaths^d^, n=	20	9	4	3	1	1	1	1	–	0.59
MMR per 100.000 hospital live births ^d^	112	184	96	89	45	51	147	262	–	

^a^Ethnicity other: Brazilian (*n* = 101, 73.7%), Caucasian (*n* = 25, 18.2%), Afro-Caribbean (*n* = 11). Missing ethnicities, *n* = 327 (1.8%)

^b^Missing ethnicities *n* = 328 with no significant differences within the variables

^c^SBR: stillbirth rate = per 1000 births > 28 weeks or > 1000 g

^d^MMR: maternal mortality rate, maternal deaths per 100.000 live births (LB), of 20 hospital maternal deaths two occurred antepartum

**Table 4:** Childbirth outcomes in different ethnicities, with Hindustani as reference group

Outcome	Odds ratio (95% confidence interval)	Adjusted Odds ratio (95% confidence interval)
**Teenage pregnancy**		**a**
Maroon	**2.1 (1.9–2.5)**	**2.1 (1.8–2.4)**
Creole	**1.4 (1.2–1.7)**	**1.4 (1.2–1.6)**
Mixed	**1.5 (1.3–1.8)**	**1.5 (1.3–1.8)**
Javanese	1.2 (1.0–1.4)	1.2 (1.0–1.4)
Indigenous	**2.7 (2.1–3.3)**	**2.6 (2.1–3.2)**
Chinese	**0.3 (0.2–0.5)**	**0.3 (0.2–0.6)**
Other	1.0 (0.6–1.8)	1.1 (0.6–1.9)
**Primiparous women**		**a**
Maroon	**0.5 (0.4–0.5)**	**0.5 (0.4–0.5)**
Creole	**0.7 (0.6–0.7)**	**0.7 (0.6–0.8)**
Mixed	**0.9 (0.8–1.0)**	**0.8 (0.8–0.9)**
Javanese	**0.8 (0.7–0.8)**	**0.7 (0.7–0.8)**
Indigenous	**0.6 (0.5–0.7)**	**0.6 (0.5–0.7)**
Chinese	0.8 (0.7–1.1)	0.8 (0.6–1.0)
Other	1.0 (0.7–1.3)	1.0 (0.6–1.3)
**Grand multiparous women**		**a**
Maroon	**10.6 (8.7–12.8)**	**14.7 (11.9–18.1)**
Creole	**4.0 (3.3–4.9)**	**4.1 (3.3–5.1)**
Mixed	**2.2 (1.7–2.8)**	**2.2 (1.7–2.9)**
Javanese	**1.8 (1.4–2.4)**	**1.7 (1.3–2.3)**
Indigenous	**6.6 (5.0–8.6)**	**9.9 (7.4–13.4)**
Chinese	**0.4 (0.2–0.9)**	**0.3 (0.1–0.8)**
Other	2.0 (1.0–4.0)	1.6 (0.7–3.4)
**Severe anemia**		**a**
Maroon	**3.7 (2.1–6.5)**	**2.4 (1.4–4.3)**
Creole	**1.9 (1.0–3.4)**	1.7 (0.9–3.0)
Mixed	1.1 (0.5–2.4)	1.8 (0.8–4.2)
Javanese	**0.2 (0.1–0.8)**	0.3 (0.1–1.3)
Indigenous	1.6 (0.5–4.8)	1.1 (0.4–3.5)
Chinese	N/A	N/A
Other	N/A	N/A
**Cesarean section**		**b**
Maroon	**0.4 (0.4–0.5)**	**0.7 (0.6–0.8)**
Creole	**0.7 (0.6–0.7)**	**0.8 (0.7–0.9)**
Mixed	0.9 (0.7–1.0)	**0.9 (0.8–1.0)**
Javanese	**0.6 (0.5–0.7)**	**0.6 (0.5–0.7)**
Indigenous	**0.5 (0.4–0.7)**	**0.8 (0.6–0.9)**
Chinese	**0.5 (0.4–0.7)**	**0.4 (0.3–0.5)**
Other	**2.0 (1.4–2.7)**	**1.7 (1.2–2.4)**
**Post partum hemorrhage**		**c**
Maroon	**1.6 (1.1–2.4)**	**1.5 (1.0–2.4)**
Creole	1.4 (0.9–2.2)	**1.4 (0.9–2.2)**
Mixed	1.4 (0.8–2.3)	1.2 (0.7–1.9)
Javanese	1.2 (0.7–2.0)	1.1 (0.7–2.0)
Indigenous	**3.2 (1.8–5.5)**	**3.0 (1.7–5.3)**
Chinese	**2.2 (1.0–4.8)**	2.1 (0.9–4.6)
Other	1.9 (0.4–7.8)	1.4 (0.3–6.0)
**Preterm birth**		**a**
Maroon	**1.3 (1.1–1.4)**	**1.2 (1.1–1.4)**
Creole	**1.2 (1.0–1.3)**	1.1 (1.0–1.3)
Mixed	1.0 (0.8–1.3)	1.0 (0.9–1.2)
Javanese	**0.8 (0.7–0.9)**	**0.8 (0.7–0.9)**
Indigenous	1.0 (0.8–1.3)	1.0 (0.8–1.3)
Chinese	**0.6 (0.4–0.8)**	**0.6 (0.4–0.9)**
Other	0.8 (0.5–1.4)	0.8 (0.4–1.4)
**Low birth weight**		**b**
Maroon	0.9 (0.8–1.0)	**0.7 (0.6–0.8)**
Creole	**0.8 (0.7–0.9)**	**0.6 (0.5–0.7)**
Mixed	**0.7 (0.6–0.8)**	**0.6 (0.5–0.7)**
Javanese	**0.5 (0.4–0.5)**	**0.4 (0.3–0.5)**
Indigenous	**0.7 (0.5–0.8)**	**0.5 (0.4–0.7)**
Chinese	**0.4 (0.3–0.6)**	**0.5 (0.3–0.8)**
Other	**0.4 (0.2–0.8)**	0.6 (0.3–1.1)
**Low APGAR score**		**c**
Maroon	**1.8 (1.4–2.3)**	**1.6 (1.2–2.0)**
Creole	**1.6 (1.3–2.1)**	**1.4 (1.0–2.0)**
Mixed	0.9 (0.7–1.3)	1.0 (0.7–1.6)
Javanese	**0.6 (0.4–0.9)**	0.7 (0.4–1.2)
Indigenous	1.2 (0.7–1.9)	1.1 (0.6–2.0)
Chinese	0.4 (0.1–1.0)	0.5 (0.1–1.7)
Other	N/A	N/A
**Late stillbirth**		**c**
Maroon	**2.1 (1.5–3.0)**	**1.7 (1.1–2.5)**
Creole	**1.5 (1.1–2.3)**	1.4 (0.9–2.1)
Mixed	0.9 (0.6–1.6)	1.0 (0.6–1.8)
Javanese	**0.5 (0.2–0.9)**	**0.4 (0.2–0.9)**
Indigenous	1.1 (0.5–2.3)	1.1 (0.5–2.5)
Chinese	0.2 (0.0–1.6)	0.3 (0.0–2.4)
Other	N/A	N/A
**Maternal death**		**d**
Maroon	3.4 (0.8–15.7)	3.3 (0.7–16.1)
Creole	1.2 (0.2–7.2)	1.2 (0.2–7.1)
Mixed	N/A	N/A
Javanese	1.7 (0.2–12.4)	1.8 (0.2–12.5)
Indigenous	2.5 (0.2–27.5)	2.5 (0.2–28.5)
Chinese	4.5 (0.4–49.4)	4.7 (0.4–53.4)
Other	N/A	N/A

**Bold = significant results.***N/A* not available

^a^Adjusted for maternal age and hospital

^b^Adjusted for maternal age, parity, hospital, birth weight, gestational age

^c^Adjusted for maternal age, parity, hospital, cesarean section, birth weight, gestational age

^d^Adjusted for maternal age, parity and hospital

MMR was highest in Maroon women with 184 (*n* = 9/4877) per 100.000 live births. In Hindustani, Javanese and mixed women MMRs were 89 (*n* = 3/3370), 51 (*n* = 1/1951) and 45 (n = 1/2240). Stillbirth rates were significantly higher for African-descendent Maroon women (25.0 per 1000 births, aOR 1.7, 95%CI 1.1–2.5) and significantly lower for Javanese women (5.7 per 1000 births, aOR 0.4 95%CI 0.2–0.9) compared to Hindustani women (11.9 per 1000 births).

## Discussion

This study provides a national overview of maternal and perinatal health in multi-ethnic, middle-income country Suriname, where no formal national perinatal registry is in place yet. Disaggregation of the perinatal data shows substantial inequities in maternal and perinatal health for specific ethnic groups.

Although Suriname is classified as an upper middle-income country, it is among countries in the Latin America and the Caribbean region with the highest maternal mortality ratio and stillbirth rate. The maternal mortality ratio of 127 maternal deaths per 100.000 live births is comparable to previous studies conducted in Suriname the past years [22, 32]. Suboptimal quality of care plays an important role in the high maternal mortality ratio in Suriname and led to different quality improvement projects such as maternal death and morbidity audits, obstetric guidelines and obstetric skills team training [22, 32, 33]. The stillbirth rate, 15.6 stillbirths per 1000 births, is second highest of the region, preceded only by Haiti (SBR 24.9), and followed by Paraguay (13.4) and Bolivia (12.9) [34]. Timing and underlying causes of stillbirths are unknown and audit and in-depth case review is necessary to understand why these babies die.

Inequity in maternal and perinatal health within countries are as great as or greater than those between countries [3–5, 35]. In the SDGs, equity in health, i.e. available and affordable high-quality health services to all, is emphasized as a priority, and this was further stressed in the 2019 Report of the Commission of the Pan American Health Organization (PAHO) on Equity and Health Inequalities in the Americas [4, 5]. Disaggregating perinatal health data can identify and target inequity within the health system. Ethnicity or race is a non-modifiable risk factor to adverse maternal and perinatal outcomes, and an important social determinant of health [4, 35]. The causal pathway of ethnical disparities is influenced partly by biological factors, such as genetic predisposition, but mostly by environmental and socio-economic mediators, such as wealth, culture, nutrition and socio-economic situation – often a reflection of underlying structural and historical drivers [3, 4]. While place of residence (urban vs. rural) is an important proxy of environmental and socio-economic factors as well as access to health care, place of residence has a smaller role in Suriname, where most pregnant women (temporarily) reside in urban areas and give birth in hospitals [20].

Biological factors of ethnical disparities seem to contribute quite strongly to infants born small for gestational age. In our study in Suriname, Hindustani women generally have more favorable socio-economic status than women of other ethnical backgrounds, yet their babies are significantly smaller [17]. An explanation for this finding is lacking, as Hindustani women are not prone to severe anemia, as seen in this study, and are generally known to have a high dietary diversity [36]. A WHO study confirms that significant differences in fetal weight are seen between ten countries, with the lowest median birth weight among Indian women, also after adjustment for maternal characteristics, gestational age and fetal sex [37]. In contrast, INTERGROWTH-21 found that when mothers’ nutritional and health needs are met and there are few environmental constraints on growth, only 3.5% of the total variability of growth was due to differences between populations [38]. It is therefore controversial to locally adjust growth charts to increase predictive performance, as they can potentially deprive smaller babies of their needs for intensified health care given that most have impaired fetal growth due to malnutrition or other environmental factors.

Giving birth during adolescence is not only a risk for adverse outcomes, but also has a negative impact on the future well-being of the mother and infant, leading to stigmatism and socio-economic consequences with school drop-out, lower employment opportunities, and a higher risk of poverty and intergenerational transmission of inequities [4, 39]. High numbers of pregnancies among teenagers were seen in Maroon (18.3%) and Indigenous women (21.4%). This results in a threefold higher adolescent birth rate for Maroons and Indigenous girls (79 and 88 per 1000 girls 15–19 years) compared to Hindustani girls (27 per 1000 girls 15–19 years). In a recently conducted nationwide survey, the adolescent birth rates for Maroon and Indigenous girls are reported even higher (124 and 99 per 1000 girls 15–19 years) [20]. While the national teenage pregnancy rate (13.8%) in Suriname is somewhat lower than in many Latin American countries (16–22%), ethnic disparities within the country are significant [40, 41]. Tailored health care services for teenagers should be made available, including prevention of teenage pregnancy with free contraception, especially geared towards the groups most at risk [24, 42].

Ethnic disparities for cesarean section rates have been observed in many low- and middle income countries [43]. This is similar to findings in Suriname, where Maroon women have the lowest cesarean section rate despite increased risks of adverse pregnancy outcomes. Hospital differences in cesarean section rates may partially reflect ethnic distributions between the hospitals.

Multiple studies in high-income countries with Caucasian majorities such as the United Kingdom, the United States, the Netherlands and different Latin American countries, demonstrated an increased risk of maternal deaths, maternal morbidity and stillbirths in ethnic minority women, such as women from African, Asian or Indigenous descent, compared to Caucasian women [12–15, 44, 45]. African-descendant Maroon women in Suriname are at two- to four-fold higher risk of stillbirth (25 stillbirths per 1000 births) compared to Asian-descendant women (12 and 6 stillbirths per 1000 births in Hindustani and Javanese women). Despite low numbers of maternal death, similar trends are found with MMRs of 184 in Maroon women compared to 89 per 100.000 live births in Hindustani women, though not statistically significant due to absolute low number of deaths.

Women from ethnic minorities, women of low socio-economic status, adolescents, migrant women and women living with HIV are particularly likely to not only have increased adverse pregnancy outcomes, but also more likely to experience disrespectful or even neglect during pregnancy and childbirth [46]. Respecful maternity care, i.e. effective communication and equal engagement of health care workers to all women, is essential in reducing disparities in pregnancy care and outcomes [47]. Dialogue, research into disparities of health care outcomes and advocacy of safe motherhood is an important public health and human rights issue.

### Limitations

A number of limitations need to be considered. First, this study only covers hospital births. Although these comprise majority of all live births in the country, women who delivered in the more deprived interior settings (mostly maroons and indigenous women), may be underrepresented. As a result, the national teenage pregnancy rate or stillbirth rate could be higher than reported in this study. Second, important explanatory or risk factors such as body mass index, smoking, level of education, level of income, residency, number of antenatal care visits and medical and obstetric history were not available. Other important indicators for quality of care, such as early neonatal mortality, timing of stillbirths and indications of caesarean sections for classification according to Robson criteria were not provided by the childbirth books [48, 49]. It is recommended that these factors are included in perinatal registries in the future.

### Recommendations

While achievement of health equity requires overarching structural changes that promote social, economic and political equality, there are specific strategies policymakers could prioritize to achieve equity in reproductive, maternal and perinatal health [3, 4]. In connection to our discussion, recommendations can be made in the following direction:

1. develop a nationwide perinatal registry that includes primary health care centers and allows for disaggregated analysis by groups at risk of inequities in health outcomes;
2. ensure that quality obstetric care along the continuum from preconception and antenatal to postpartum, including safe abortion services, is accessible to all equitably;
3. monitor the quality of care, including auditing of maternal and perinatal mortality, stillbirths, severe maternal morbidity and caesarean sections;
4. provide free contraception and adolescent programs for sexual and reproductive health;
5. strenghten community based outreach and improve health literacy of women; and
6. address the structural drivers and conditions of daily life which determine equity and a dignified life.

## Conclusion

This is the a nationwide comprehensive overview of maternal and perinatal health status in Suriname, a middle-income country in South America and shows substantial inequities in maternal and perinatal health by ethnicity. African descendent Maroon women experienced the highest risk of adverse outcomes (maternal mortality, stillbirth, increased preterm birth, low apgar score). Hindustani women have lower risk on adverse outcomes, yet give birth to smaller babies and give birth by cesarean section most frequently. Disaggregating perinatal health data can identify and help target inequity within the health system.

## Supplementary information

**Additional file 1.** Visual summary of data availability per hospital.

**Additional file 2.** Differences in maternal and neonatal characteristics between hospitals in Suriname.

## Data Availability

The datasets used and/or analyzed during the current study are available from the corresponding author on reasonable request.

## Abbreviations

ANC: Antenatal Care
LB: Live birth
MDG: Millennium Development Goals
MICS: Multiple Indicator Cluster Survey
MMR: Maternal mortality ratio
(a)OR: (Adjusted) odds ratio
PAHO: Pan American Health Organization
PPH: Post-partum hemorrhage
SIP: Perinatal Information System
SBR: Stillbirth Rate
SDG: Sustainable Development Goals
WHO: World Health Organization
