# Supplementary figures and images for: Childbirth outcomes and ethnic disparities in Suriname: a nationwide registry-based study in a middle-income country
Source: Reprod Health. 2020 May 7;17:62. doi: 10.1186/s12978-020-0902-7 (PMC7206667; doi:10.1186/s12978-020-0902-7)

Supplementary File 1 Visual summary of data availability per hospital (I-V)

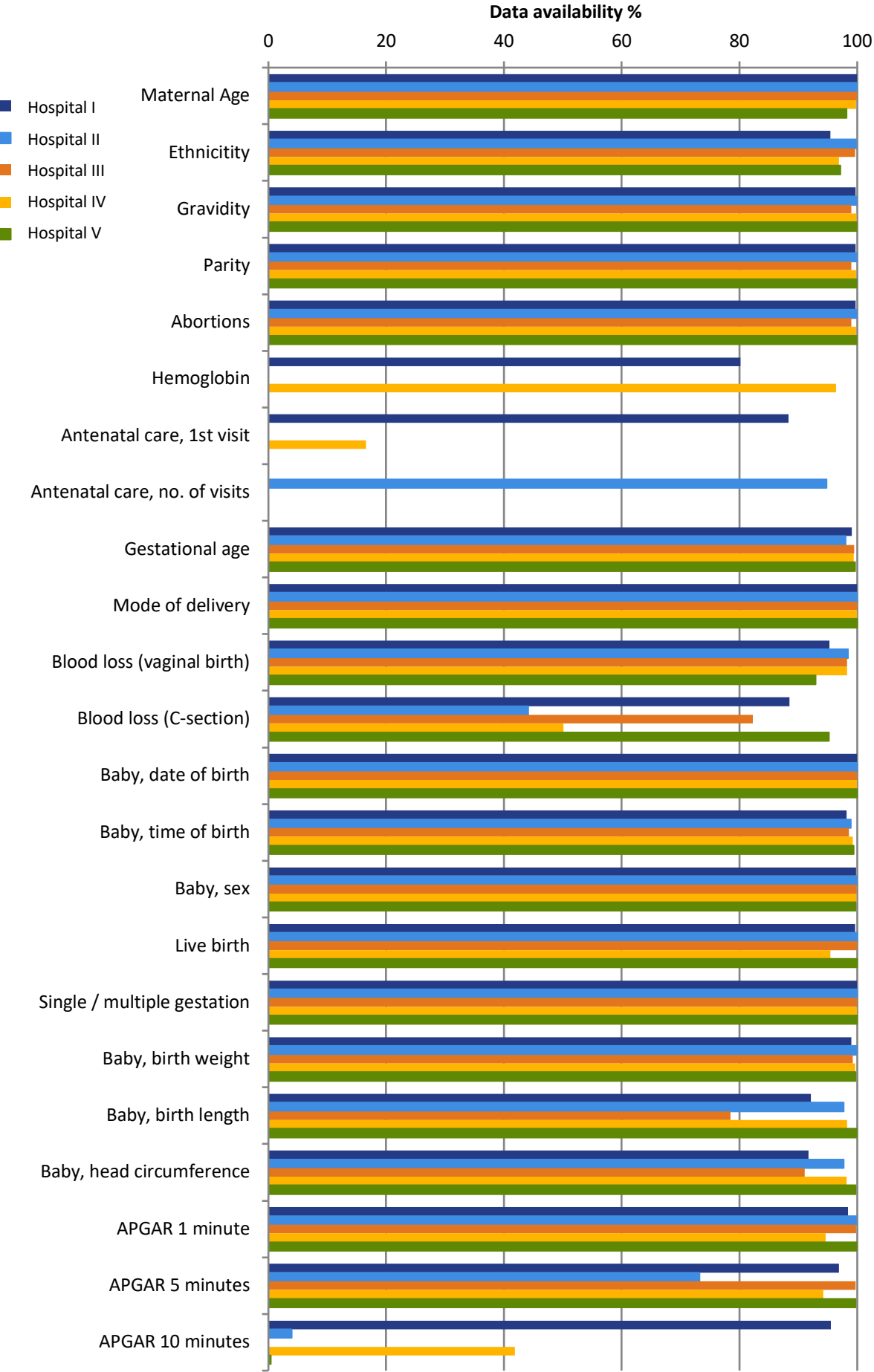

Supplement: Supplementary file 1 — Additional file 1. Visual summary of data availability per hospital. [file 12978_2020_902_MOESM1_ESM.pdf]
