# Supplementary material for: Childbirth outcomes and ethnic disparities in Suriname: a nationwide registry-based study in a middle-income country
Source: Reprod Health. 2020 May 7;17:62. doi: 10.1186/s12978-020-0902-7 (PMC7206667; doi:10.1186/s12978-020-0902-7)
Supplement: Supplementary file 2 — Additional file 2. Differences in maternal and neonatal characteristics between hospitals in Suriname. [file 12978_2020_902_MOESM2_ESM.pdf]

Supplementary file 2 Differences in maternal and neonatal characteristics between hospitals in Suriname (2016-2017)

	Hospital I n = (%)	Hospital II n = (%)	Hospital III n = (%)	Hospital IV n = (%)	Hospital V n = (%)	p value
**Deliveries**	4380 (23.9)	5070 (27.7)	5089 (27.8)	3034 (16.6)	717 (3.9)	<0.05
2016	*2272 (24.7)*	*2418 (26.3)*	*2633 (28.6)*	*1531 (16.6)*	*348 (3.8)*	
2017	*2108 (23.2)*	*2652 (29.2)*	*2456 (27.0)*	*1503 (16.5)*	*369 (4.1)*	
**Teenage pregnancy**						
< 20 years	782 (17.9)	567 (11.2)	835 (16.4)	222 (7.3)	112 (15.9)	
20 – 35 years	3165 (72.6)	3762 (74.2)	3728 (73.3)	2446 (80.7)	545 (77.4)	<0.05
≥ 36 years	414 (9.5)	741 (14.6)	522 (10.3)	363 (12.0)	47 (6.7)	
**Ethnicity**						
Maroon	1758 (41.9)	1254 (24.8)	1746 (34.4)	189 (6.5)	3 (0.4)	
Creole	1138 (27.1)	1342 (26.6)	1078 (21.2)	614 (20.9)	45 (6.5)	
Hindustani	700 (16.7)	940 (18.5)	808 (15.9)	554 (18.3)	393 (54.8)	
Mixed	168 (4.0)	654 (12.9)	735 (14.5)	579 (19.7)	117 (16.8)	
Javanese	251 (6.0)	605 (12.0)	422 (8.3)	594 (20.2)	76 (10.9)	< 0.05
Inheems	166 (4.0)	170 (3.4)	247 (4.9)	54 (1.8)	45 (6.5)	
Chinees	14 (0.3)	62 (1.2)	29 (0.6)	260 (8.8)	16 (2.3)	
Other	1 (0.0)	25 (0.5)	14 (0.3)	96 (3.3)	1 (0.1)	
**Parity**						
0	1226 (28.1)	1942 (38.7)	1502 (29.5)	1295 (42.7)	278 (38.8)	
1 - 3	2333 (53.4)	2470 (49.2)	2770 (54.5)	1598 (52.7)	382 (53.4)	<0.05
≥ 4	810 (18.5)	609 (12.1)	815 (16.0)	138 (4.6)	56 (7.8)	
**Anaemia**						
Moderate	1612 (45.9)	N/A	N/A	728 (31.1)	N/A	<0.05
Severe	138 (3.9)	N/A	N/A	15 (0.5)	N/A	
**Mode of delivery**						<0.05
Spontaneous	3595 (82.1)	3835 (75.6)	3733 (73.4)	1933 (63.7)	554 (77.3)	
Vacuum	12 (0.3)	135 (2.7)	44 (0.9)	2 (0.1)	38 (5.3)	
Cesarean	773 (17.6)	1100 (21.7)	1312 (25.8)	1099 (36.2)	125 (17.4)	<0.05
**Post partum hemorrhage**						
500 - 999 mL	204 (4.9)	253 (5.3)	412 (9.6)	108 (4.4)	54 (8.1)	
≥ 1000 mL	59 (1.4)	67 (1.4)	90 (2.1)	24 (1.0)	16 (2.4)	<0.05
**Gestational age**						
< 28 weeks	113 (2.6)	44 (0.9)	39 (0.8)	15 (0.5)	6 (0.8)	
32 – 36 weeks	669 (15.4)	515 (10.2)	729 (14.6)	327 (10.8)	72 (10.1)	<0.05
≥ 37 weeks	3558 (82.0)	4484 (88.9)	4218 (84.6)	2673 (88.7)	636 (89.1)	
**Total babies born**	4444	5138	5147	3058	717	
Live Births	4281 (96.3)	5048 (98.2)	5052 (98.2)	3031 (99.1)	705 (98.3)	<0.05
Stillbirths ≥22 weeks or 1000 g	163 (3.7)	90 (1.8)	94 (1.8)	27 (0.9)	12 (1.7)	
**Birth weight**						
< 2500 grams	917 (20.8)	625 (12.3)	789 (15.3)	344 (11.3)	99 (13.8)	
2500 – 4000 grams	3373 (76.7)	4296 (84.3)	4234 (82.3)	2580 (84.7)	586 (82.0)	<0.05
≥ 4000 grams	109 (2.5)	176 (3.5)	120 (2.3)	123 (4.0)	30 (4.2)	
**APGAR score 5 minutes**						
Below 7	273 (6.3)	164 (3.2)	133 (3.5)	48 (1.7)	30 (4.2)	<0.05
**Stillbirths ≥ 28 weeks**						
n=	109	69	74	22	11	
SBR per 1000 births [1]	25.6	13.7	14.7	7.3	15.5	<0.05
**Maternal deaths**						
n=	8	3	4	5	-	
MMR per 100.000 live births [2,3]	186.9	59.4	79.1	165.0	-	<0.05

[1] SBR: stillbirth rate = per 1000 births ≥ 28 weeks or ≥ 1000 grams
[2] MMR: maternal mortality rate, maternal deaths per 100.000 live births (LB), hospital is location of death, n=5 occurred at home or in primary health care services.
[3] Maternal deaths: one deaths in hospital I was a delivery from hospital II and one was a home delivery, one death in hospital II died during transport to the hospital, one death in hospital IV was a woman who gave childbirth in hospital I.
